# National Nutrition Surveys Applying Dietary Records or 24-h Dietary Recalls with Questionnaires: A Scoping Review

**DOI:** 10.3390/nu15224739

**Published:** 2023-11-09

**Authors:** Emiko Okada, Makiko Nakade, Fumiaki Hanzawa, Kentaro Murakami, Mai Matsumoto, Satoshi Sasaki, Hidemi Takimoto

**Affiliations:** 1 The Health Care Science Institute, 3-2-12 Akasaka, Minato-ku, Tokyo 107-0052, Japan; 2 Department of Nutritional Epidemiology and Shokuiku, National Institute of Biomedical Innovation, Health and Nutrition, Kento Innovation Park, NK Building, 3-17 Senrioka Shinmachi, Settsu-shi 566-0002, Japan; 3 Department of Food Science and Nutrition, University of Hyogo, 1-1-12 Shinzaike-Honcho, Himeji-shi 670-0092, Japan; 4 Research Institute for Food and Nutritional Sciences, 1-1-12 Shinzaike-Honcho, Himeji-shi 670-0092, Japan; 5 Department of Social and Preventive Epidemiology, School of Public Health, University of Tokyo, Tokyo 113-0033, Japan

**Keywords:** dietary records, 24-h dietary recall, questionnaires, national survey, scoping review

## Abstract

Development of an accurate and efficient dietary method is required for national nutrition surveys. Some countries conduct dietary surveys and combine 24-h dietary records or 24-h dietary recalls with dietary questionnaires. This scoping review aimed to summarize studies that used results from national surveys that combined detailed dietary surveys (dietary records or 24-h dietary recall) and dietary questionnaires and identify the purpose of combining the two methods. The PubMed database and manual searches were used for the literature review. We extracted 58 articles from 16 national nutrition surveys from 14 countries. Most studies used 24-h dietary recall for detailed dietary surveys and the food frequency questionnaire (FFQ) or food propensity questionnaire (FPQ) for questionnaire surveys. Among 37 studies from eight countries, the purpose of combining the two dietary survey methods was to estimate energy and nutrient intakes from detailed dietary surveys and habitual food intake from questionnaires. These findings are useful as a reference when introducing new dietary survey methods in future national nutrition surveys.

## 1. Introduction

National nutrition surveys are conducted worldwide to assess people’s health status and nutritional intake [1,2]. Results from many national dietary surveys are used to monitor the population’s health and nutritional status. Furthermore, they are utilized to develop national nutritional policies and formulate dietary guidelines [3,4,5,6,7].

Dietary survey methods applied in national surveys are similar to those used in various epidemiological studies, such as dietary records, 24-h dietary recalls, food frequency questionnaires (FFQs), and dietary history methods [8]. Some national surveys that conduct dietary surveys combine 24-h dietary recall and FFQs or the food propensity questionnaire (FPQ) (the National Health and Nutrition Examination Survey (NHEANS) from 1999 to 2006 in the United States [9], the Korea National Health and Nutrition Examination Survey (KNHANES) from 2007 to 2018 [10], and the Slovenian national dietary survey (SI.Menu 2017/18) [11]). Detailed dietary surveys, such as dietary records and 24-h dietary recall, can estimate participants’ nutrient intake. However, these methods impose a heavy burden on the participants and investigators [8]. Conversely, although it is more difficult to estimate the absolute value of nutrient intake via FFQs than through detailed surveys, it can be used in large-scale surveys owing to it being less burdensome and simpler for participants. In addition, it can assess habitual intake [8]. Hence, combining 24-h dietary recall and questionnaire methods may compensate for each of the methods’ disadvantages. However, the purpose of these two dietary survey methods remains unclear.

Therefore, this scoping review aimed to summarize studies that used results from national surveys that combined detailed dietary surveys, such as dietary records or 24-h dietary recall, with questionnaires and identify the purpose of combining the two methods.

## 2. Materials and Methods

This review protocol was designed based on the methodology and guidance for conducting systematic scoping reviews [12] and the Preferred Reporting Items for Systematic reviews and Meta-Analyses extension for Scoping Reviews [13] and was planned in advance.

### 2.1. Search Strategy

Articles published from inception to 20 October 2022 were searched via the PubMed database. Literature containing the terms “dietary record” or “24-h dietary recall” for detailed dietary surveys, as well as “questionnaire” and “national survey,” were searched for. The following search terms were employed, determined based on Mesh terms: (“food record”[tiab] OR “food records”[tiab] OR “diet record”[tiab] OR “diet records”[tiab] OR “food diary”[tiab] OR “food diaries”[tiab] OR “dietary record”[tiab] OR “dietary records”[tiab] OR “recall method”[tiab] OR “recall”[tiab] OR “recalls”[tiab] OR “diet records”[MeSH]) AND (“food frequency questionnaire”[tiab] OR “FFQ”[tiab] OR “FFQs”[tiab] OR “food frequency method”[tiab] OR “diet history questionnaire”[tiab] OR “diet history method”[tiab] OR ((“diet”[tiab] OR “Food”[tiab]) AND “Questionnaire”[tiab]))) AND (“national”[tiab] OR ”nationwide”[tiab]). The search terms were determined after repeated consideration by the review team (E.O., M.N., M.M., K.M., S.S., and H.T.).

### 2.2. Inclusion and Exclusion Criteria

This review included articles that met the inclusion criteria: (1) peer-reviewed original articles, (2) studies written in English, (3) studies conducted with the same person and using both a detailed dietary survey of the dietary record or 24-h dietary recall and a questionnaire, and (4) studies conducted via data from a national survey. We excluded the following references: (1) review articles and conference proceedings, (2) studies conducted for study purposes, and (3) survey methodologies.

### 2.3. Selection of Articles

First, the titles and abstracts of the identified articles underwent screening in accordance with the eligibility criteria (E.O., M.N., and F.H.). Second, full texts were assessed for eligibility, and articles for review were extracted (E.O., M.N., and F.H.). Additionally, potentially relevant articles were manually searched and additional articles were identified. All of the articles were screened or independently reviewed by at least two researchers. Any discrepancies were discussed and resolved through consensus or with the involvement of another reviewer, if necessary (H.T. and S.S.).

### 2.4. Data Extraction

Data were extracted and summarized into a standardized tabular format (Excel sheet) by two independent reviewers. The extracted information included (1) authors, (2) publication year, (3) country, (4) survey name, (5) study year, (6) population and sample size, (7) aims, (8) detailed dietary survey method (dietary record or 24-h dietary recall), (9) dietary questionnaire, (10) how to use the dietary survey results from the detailed dietary method and dietary questionnaire, and (11) limitations of the dietary survey methods (if applicable).

## 3. Results

### 3.1. Study Selection

**Figure 1:**
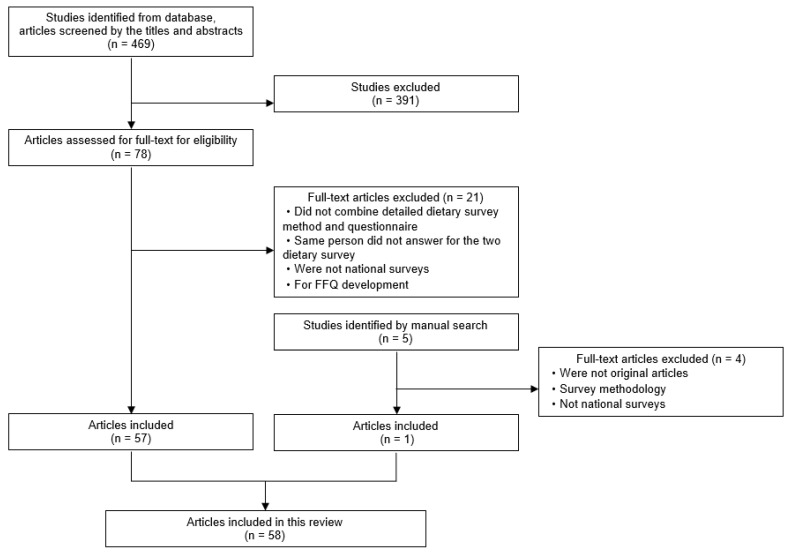
Flowchart of the selection process.

A total of 469 articles were extracted. Figure 1 shows the flowchart of the literature selection process. After 391 articles that did not meet the inclusion criteria were excluded during the initial title and abstract screening, 78 articles underwent full screening. In the full-text screening, 21 articles were excluded, including studies that did not combine detailed dietary survey methods and questionnaires, studies that did not conduct the two dietary survey methods for the same individuals, studies that did not use the data from national surveys, and studies employing a survey methodology (FFQ development not examining the validation of the FFQ), based on the exclusion criteria. Furthermore, one article was identified via a manual search [14]. A total of 58 articles were included.

### 3.2. Study Characteristics

**Figure 2:**
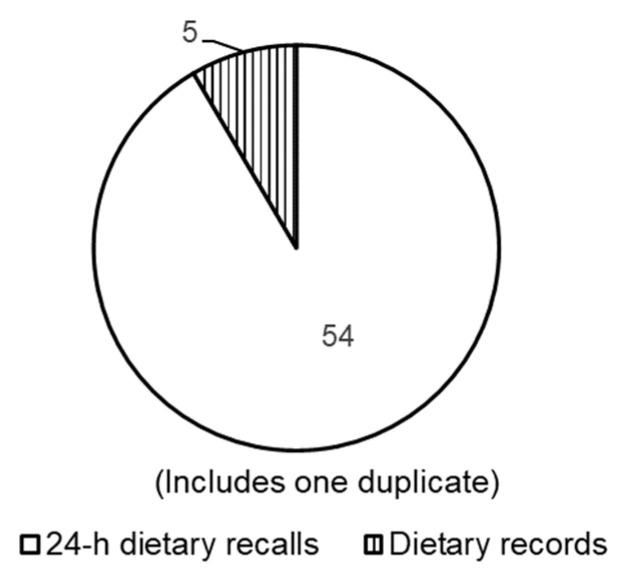
Breakdown of the detailed dietary surveys.

**Figure 3:**
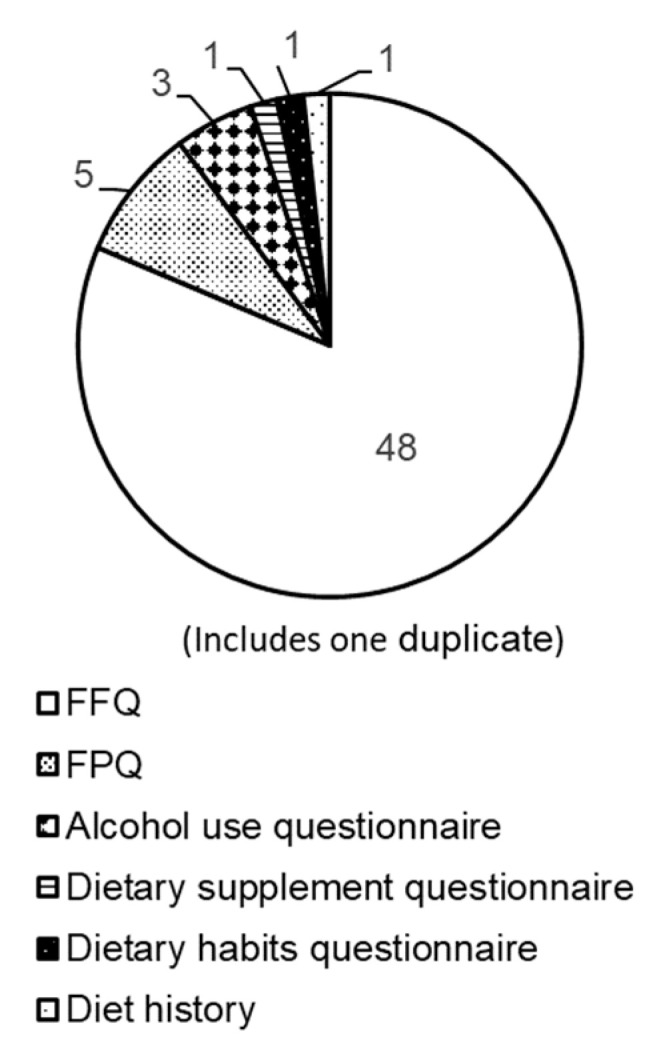
Breakdown of the dietary questionnaire surveys.

The included studies used data from the national surveys in 14 countries: the United States [15,16,17,18,19,20,21,22,23,24,25,26,27,28,29,30,31,32], Australia [33], Cameroon [34], Korea [14,35,36,37,38,39,40,41,42,43,44,45,46,47,48], Greece [49,50,51], Sweden [52,53], Slovenia [54,55], Taiwan [56,57,58,59], Germany [60,61,62], New Zealand [63], Norway [64], Belgium [65,66,67,68], South Africa [69], and Mexico [70,71]. Table 1 shows the characteristics of the included studies. Of the 58 articles, 18 studies used data from the NHEANS in the United States [15,16,17,18,19,20,21,22,23,24,25,26,27,28,29,30,31,32] and 15 used data from the KNHANES in Korea [14,35,36,37,38,39,40,41,42,43,44,45,46,47,48]. Some countries had two national surveys: the National Health and Nutrition Survey (HYDRIA) [49,50] and the Hellenic National Nutrition and Health Survey [51] in Greece and the Children’s Nutrition Survey to Record Food Consumption (KiESEL) [60] and the German National Nutrition Survey II [61] in Germany. Participants’ ages ranged from 2 months to >65 years, and the sample sizes ranged from 64 to 27,656. In total, 54 studies [14,15,16,17,18,19,20,21,22,23,24,25,26,27,28,29,30,31,32,33,34,35,36,37,38,39,40,41,42,43,44,45,46,47,48,49,50,51,54,55,56,57,58,59,61,62,63,65,66,67,68,69,70,71] from 12 countries used 24-h dietary recall as a detailed dietary survey in national surveys (Figure 2). Dietary records were used in the KiESEL in Germany [60], Belgium (age 3–9 years) [66], Sweden (web-based food records) [52,53], and Norway (weighed method) [64]. The qualitative/semi-quantitative FFQ was used in 48 studies [14,16,17,19,20,21,22,23,24,26,27,28,29,30,31,32,33,34,35,36,37,38,39,40,41,42,43,44,45,46,47,48,50,52,53,56,57,58,59,62,64,65,66,67,68,69,70,71] for dietary surveys via questionnaires (Figure 3). The FPQ was used in five recent studies [49,51,54,55,60]. Studies in the United States [15,18,25], Greece [49], and Taiwan [59] also used questionnaires enquiring about alcohol consumption or supplement use.

### 3.3. Limitations of Dietary Survey Methods

In total, 10 studies reported that 24-h dietary recall from one day could not assess habitual dietary intake [14,21,23,28,30,31,35,45,47,63] (Table 1). Overall, the limitations indicated the possibility of measurement errors and recall bias in under-/over-reporting. 

### 3.4. Purpose of Combined Detailed Dietary Survey of Dietary Record or 24-h Dietary Recall and Questionnaire

**Figure 4:**
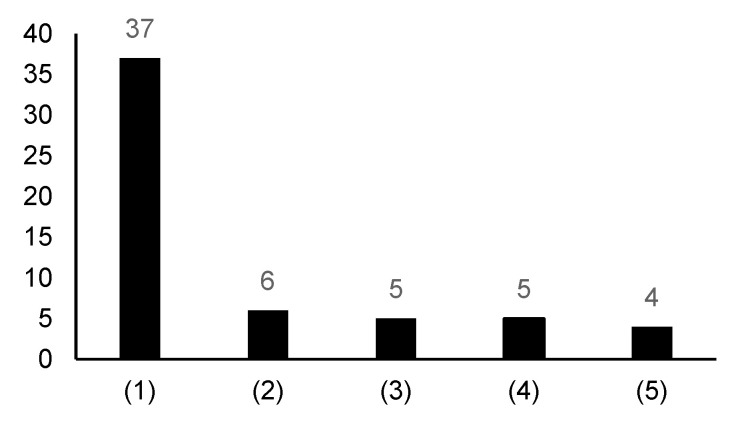
The number of articles covering each main purpose of conducting a dietary record or 24-h dietary recall combined with a questionnaire: (1) to estimate the intake of energy, nutrients, and foods or calculate the dietary scores from the results of the detailed dietary survey and evaluate habitual foods intake frequency and intake/non-intake from a questionnaire survey [14,15,17,18,19,20,21,22,23,26,27,28,29,30,31,32,34,35,36,37,38,40,41,42,43,44,45,46,47,48,49,56,57,58,63,65,69]; (2) to use the results of the questionnaire survey to complement the intake of energy, nutrients, and foods obtained from the results of detailed dietary surveys [24,33,50,54,55,66]; (3) to compare the results of the detailed dietary survey to verify the validity of the questionnaire survey [51,62,64,70,71]; (4) to evaluate the intake status of infrequently consumed foods from the questionnaire survey [25,52,53,60,68]; and (5) to compare intakes of energy, nutrients, and foods estimated from the detailed dietary survey and questionnaire survey [16,39,59,61].

Figure 4 shows a summary of the main purposes of conducting a detailed dietary survey of the dietary record or 24-h dietary recall combined with a questionnaire survey: (1) to estimate the intake of energy, nutrients, and foods or calculate dietary scores from the results of the detailed dietary survey and evaluate habitual food intake frequency and intake/non-intake from a questionnaire survey (37 studies from eight countries) [14,15,17,18,19,20,21,22,23,26,27,28,29,30,31,32,34,35,36,37,38,40,41,42,43,44,45,46,47,48,49,56,57,58,63,65,69]; (2) to use the results of the questionnaire survey to complement the intake of energy, nutrients, and foods obtained from the results of detailed dietary surveys (six studies from five countries) [24,33,50,54,55,66]; (3) to compare the results of the detailed dietary survey to verify the validity of the questionnaire (five studies from four countries) [51,62,64,70,71]; (4) to evaluate the intake status of infrequently consumed foods from the questionnaire survey (wild game, animal internal organs, seafood, supplements, specific foods) (five studies from four countries) [25,52,53,60,68]; and (5) to compare intakes of energy, nutrients, and food estimated from the detailed dietary survey and questionnaire survey (four studies from four countries) [16,39,59,61]. One study did not provide any information as to why the two dietary survey methods were combined [67].

## 4. Discussion

This scoping review summarized 58 studies that used results from national surveys that combined detailed dietary surveys, such as dietary records or 24-h dietary recall, and questionnaires. We found 16 nutritional surveys in 14 countries that used a detailed dietary survey combined with a questionnaire. Most studies used the 24-h dietary recall and the FFQ or FPQ as a detailed dietary survey and questionnaire survey, respectively. More than half estimated energy and nutrient intakes from the detailed dietary surveys and evaluated habitual food intake from the questionnaire. 

Although dietary records or 24-h dietary recall could estimate food, energy, and nutrient intake from information regarding the foods consumed on the survey day, their use in long-term surveys was difficult owing to the heavy burden on the participants and investigators [8]. The included studies reporting that a single day of dietary recall could not assess habitual intake, which was a limitation of the dietary survey [14,21,23,28,30,31,35,45,47,63]. Conversely, surveys that used questionnaires, such as the FFQ, were limited in the number of foods to be investigated; however, it was possible to evaluate participants’ habitual food intake [8]. In addition, in some studies, questionnaires captured the frequency of consumption of foods that were not frequently consumed and that did not emerge in detailed dietary surveys over a short period of time [25,52,53,60,68]. The FFQ is also useful for assessing food safety and exposure to chemical substances and environmental pollutants, which are important for food sanitation administration. The KiESEL study, eligible for young children in Germany, was conducted to estimate the exposure to substances in food, such as contaminants, pesticides, or microbial risks, as required for health assessment [60]. Since detailed dietary and questionnaire surveys had different strengths and weaknesses, many included studies were used to compensate for the disadvantages of each dietary survey method and take advantage of their benefits. 

To use both dietary methods, it may be necessary to devise ways to reduce the burden on the participants and survey staff. The introduction of a 24-h dietary recall system (such as Intake24 in the United Kingdom [72] or the GloboDiet software adapted to each country in European countries and Korea [73]) that allows participants to enter their dietary intake on a web-based basis and automatically calculate nutrient and food intakes based on the input results could be beneficial. Among the included studies, Riksmaten assessed dietary intake in Sweden via a web-based food record where participants entered their own dietary information [52,53]. Another method to reduce the burden was to determine the calibration coefficients from a regression model and compare the nutrient intake estimated from the FFQ with the intake calculated more accurately from a 24-h dietary recall [74]. Bassett et al. [74] conducted a study using data from a follow-up of the Melbourne Collaborative Cohort Study in Australia. A method to assess population nutrient intake via calibration coefficients would reduce the number of participants for the 24-h dietary recall. Furthermore, many participants may only need to answer the FFQ. However, our review did not include articles that determined the calibration factors from two dietary survey methods. Examining ways to determine the correction coefficients from the two dietary survey methods in national surveys worldwide is necessary. 

National nutrition surveys are important to conduct without interruption, as they monitor the health and nutritional status of the population [1,2] and serve as the basis for national nutrition policy and dietary guidelines [3,4,5,6,7]. Although the same dietary survey methods should be used for continuous assessment, new survey methods should also be considered in light of current trends and global conditions, such as the COVID-19 pandemic. In countries that use only one survey method, conducting the FFQ or FPQ, including web-based versions, could be considered for participants with a large-enough sample size, and then the 24-h dietary recall could be implemented for some of the participants. As our results show, the values estimated from the 24-h dietary survey can be used to adjust energy, nutrient, and food intakes obtained from the FFQ by using the two survey methods together [24,33,50,54,55,66]. However, the skills of the investigators are critical to conducting a face-to-face survey, such as the 24-h dietary recall or dietary record. There is a need to train skilled nutritionists and provide a certain training period at the implementing agency or other institutions in a systematic manner to improve the accuracy and standardization of the survey methodology, as is carried out in several countries [75,76,77]. Moreover, considering that the 24-h recall method may have difficulty estimating seasoning intake, one might contemplate using the dietary record method to assess seasoning intake [78]. In addition, to accurately assess nutrient intake, the questionnaire survey should include items related to the use of dietary supplements as well as the frequency of food intake [47,55]. If a more accurate measurement of nutrient intake is desired, it may be worth considering the introduction of bioindicators that do not rely on participants’ memory. Dietary survey methods in national surveys need to be consider based on the national characteristics, dietary habits, dietary culture, and the feasibility of the survey.

The main strength of this scoping review was that we extracted the purposes of conducting a dietary record or 24-h dietary recall combined with a questionnaire survey via results from national surveys, following scoping review guidelines. We suggested improvements that are needed when employing both 24-h dietary recalls and questionnaires, taking advantage of the strengths and compensating for the weaknesses of each survey method. The results will be useful for national nutrition surveys in countries worldwide and for examining new dietary survey methods consistent with the changing times. However, this scoping review has several limitations. First, there may have been other national surveys that combined the two dietary survey methods that were not included. Some national surveys combined 24-h dietary recall or dietary records and the FFQ; however, they were not extracted from the search. These include the Chinese National Nutrition and Health Surveillance (CNNHS) from 2010 to 2013 [79], the Third Individual and National Food Consumption Survey (INCA3) from 2014 to 2015 in France [80], and the National FinHealth Study in 2017 in Finland [6]. Regarding these three national surveys [6,79,80], we searched the original articles that used the data from the survey results. However, these studies used the results of only one dietary survey method. Second, articles that used the results of only one of the two dietary survey methods (either dietary record or 24-h dietary recall and questionnaire) conducted simultaneously on the participants were not included. Therefore, the results based on combining the two dietary survey methods may have been biased. Finally, although we used various search terms and manual searches, we may not have captured all relevant articles. In addition, we used only one database and limited the literature search to the English language.

## 5. Conclusions

This scoping review identified the purpose of combining a detailed dietary survey of dietary record or 24-h dietary recall with a questionnaire via results from national surveys. Most studies used 24-h dietary recall for detailed dietary surveys and the FFQ or FPQ for questionnaire surveys. More than half of the included studies estimated nutrient intake from the detailed dietary surveys and evaluated habitual food intake from questionnaires. Our results are useful as a reference for introducing new dietary survey methods into future national nutrition surveys.

## Figures and Tables

**Table 1:** Characteristics of the included studies.

Author and Publication Year	Country	Survey Name	Study Year	Study Population and Sample Sizes	Study Aims	Detailed Dietary Survey Method of Dietary Record or 24-h Dietary Recall	Dietary Survey Method Using Questionnaire	How to Use the Dietary Survey Results from Dietary Record or 24-h Dietary Recall and Questionnaire	Limitations of the Dietary Survey Methods
Kanellou et al., 2022 [49]	Greece	National Health and Nutrition Survey (HYDRIA)	2013–2014	4011 adults aged ≥18 years (1873 males and 2138 females)	To examine the relationship between dietary supplement intake and lifestyle, health status, and eating habits	Two 24-h dietary recalls	The FPQ and supplement use questionnaire	24-h dietary recall: estimate dietary supplement use (frequency and quantity) FPQ: evaluate dietary supplement use (frequency and quantity) Questionnaire: evaluate dietary supplement use (frequency and quantity)	NA
Joseph et al., 2022 [15]	US	NHANES	2017–2018	2320 adults aged ≥18 years	To examine the association between alcohol consumption thresholds and macronutrient composition, energy intake, and anthropometric measures	Two 24-h dietary recalls	Alcohol use questionnaire	24-h dietary recall: estimate nutrient intake Questionnaire: evaluate frequency and amount of alcohol consumption	There is a lack of specific data on macronutrient composition in alcoholic and non-alcoholic diets and the exact energy intake from different types of alcohol.
Gregorič et al., 2022 [54]	Slovenia	SI.Menu 2017/18	2017–2018	364 adults aged 18–64 years (173 males and 191 females) and 416 older adults aged 65–74 years (213 males and 203 females)	To assess Slovenian adults and older adults’ dietary habits regarding food consumption and energy and macronutrient intakes	Two non-consecutive 24-h dietary recalls	FPQ	Both 24-h recalls and FPQs were used to estimate usual food and nutrient intakes. 24-h dietary recall: estimate usual daily dietary intake of foods and nutrients FPQ: evaluate usual daily dietary intake of foods and nutrients	The 24-h recall method is susceptible to reporting bias. Slow processing speed of upgraded OPEN software for SI.Menu projects may affect food matching and accuracy. Open-ended food selection and web-based recalls may lead to variations of the results. Recipes from the same country may differ due to diverse methods and ingredients.
Nowak et al., 2022 [60]	Germany	The Children’s Nutrition Survey to Record Food Consumption (KiESEL)	2014–2017	1104 children aged 6 months to 5 years (560 boys and 544 girls)	To update German children’s consumption data for use in exposure (e.g., contaminants, pesticides or microbial risks) assessment	Three consecutive days and an unrelated fourth day of dietary records	FPQ	FPQ: evaluate frequency of consumption of foods that were rarely eaten, including offal of various animals or foods, and various teas and herbal infusions	NA
Oviedo-Solís et al., 2022 [70]	Mexico	Mexican National Health and Nutrition Survey (Ensanut 2012)	2011–2012	217 children aged 5–11 years and 165 adolescents aged 12–19 years	To evaluate the relative validity of a semi-quantitative FFQ compared with two 24-h dietary recalls for estimating dietary intake in each NOVA food group in Mexican children and adolescents	Two 24-h dietary recalls	Semi-quantitative FFQ	Evaluation of agreement of energy and nutrient intakes estimated by the FFQ and the 24-h recall method 24-h dietary recall: estimate energy and nutrient intakes FFQ: estimate energy and nutrient intakes	Since not all cooking methods were covered, it is likely that some foods were misclassified among the NOVA groups. In some cases, it was unclear whether the food was prepared at home or commercially available.
Martimianaki et al., 2022 [50]	Greece	National Health and Nutrition Survey (HYDRIA)	2013–2014	4011 adults aged 18–94 years (1873 males and 2138 females)	To assess the level of adherence to the traditional Greek Mediterranean diet by examining the food and macronutrient intake of the Greek population	Two non-consecutive 24-h dietary recalls	Non-quantitative FFQ	The frequency of foods from the FFQ was incorporated into the model to supplement the food intakes from the 24-h dietary recalls. 24-h dietary recall: estimate food intakes and the usual distribution intake of food groups and subgroups FFQ: evaluate frequency of foods	It was not possible to differentiate between refined grains and whole grains in the cereal and products food groups.
Jung et al., 2022 [35]	Korea	KNHANES	2016	3189 adults aged 19–64 years (1215 males and 1974 females)	To evaluate the performance of the FFQ to estimate the contribution of NOVA groups in an individual’s diet compared to a single 24-h dietary recall, with a particular focus on ultra-processed foods	One 24-h dietary recall	Dish-based semiquantitative FFQ	Four NOVA groups were categorized and mean differences, correlation coefficients, and co-classifications between FFQ and 24-h recall for energy and nutrient intakes in each group were calculated. 24-h dietary recall: estimate all food and beverage intakes FFQ: evaluate all food and beverage intakes	Misclassification of NOVA groups, including UPF, was possible because the FFQ was not designed for the NOVA system. Findings may not apply to the food-based FFQ, and neither the single 24-h recall nor the FFQ was the gold standard for dietary intake.
Shim et al., 2021 [36]	Korea	KNHANES	2008–2011	FFQ: 13,132 adults (5407 males and 7725 females), 24-h dietary recall: 13,366 adults (5522 males and 7844 females) aged ≥19 years	To analyze the relationship between egg consumption, body composition, and serum cholesterol levels	One 24-h dietary recall	Non-quantitative FFQ	24-h dietary recall: assess egg intake (not consumed or consumed) FFQ: evaluate frequency of egg consumption	Questionnaire-based data may have recall bias.
Pedroni et al., 2021 [65]	Belgium	Belgian National Food Consumption Survey (BNFCS—2014)	2014–2015	1158 adults aged 18–64 years	To estimate differences in costs per diet quality and identify sociodemographic characteristics associated with cost differences in adult diets	Two non-consecutive 24-h dietary recalls	Qualitative FFQ	24-h dietary recall: calculate the traditional Mediterranean Diet Score, Healthy Diet Indicator, and average daily dietary costs FFQ: calculate the traditional Mediterranean Diet Score	The food group definitions may be outdated, lacking differentiation between whole and refined grains and not accounting for ultra-processed foods.
Hribar et al., 2021 [55]	Slovenia	SI.Menu 2017/18	2017–2018	1248 participants (468 adolescents (238 males and 230 females) aged 10–17 years, 364 adults (173 males and 191 females) aged 18–64 years, 416 older adults (213 males and 203 females) aged 65–74 years)	To estimate vitamin D intake, identify food groups that contribute significantly to vitamin D intake, and predict the impact of a hypothetical milk fortification mandate	Two non-consecutive 24-h dietary recalls	FPQ	Vitamin D intake was estimated using data from the 24-h recalls and FPQ. 24-h dietary recall: estimate vitamin D intake and vitamin D food intakes of usual diet using the Multiple Source Method FPQ: evaluate vitamin D intake and vitamin D food intakes of usual diet using the Multiple Source Method	The FPQ data did not include eating frequency for some important vitamin D sources, like eggs. Vitamin D content was estimated from a food composition database, not laboratory analysis. Only dietary vitamin D intake was considered, excluding pharmaceuticals and dietary supplements.
Mayasari et al., 2021 [56]	Taiwan	Pregnant NAHSIT	2017–2019	1430 pregnant women aged ≥15 years	To investigate the relationship between food and nutrient intake and serum hepcidin levels for iron status on a population scale	One 24-h dietary recall	Interviewer-administrated nonquantitative FFQ	24-h dietary recall: estimate nutrient intake FFQ: evaluate food intake frequency	A single 24-h recall dataset was utilized. Self-reported dietary data may lead to recall bias and underreporting of energy intake, particularly among overweight or obese individuals in late pregnancy. Pregnant women might overreport dietary intake in the FFQ.
Kim et al., 2021 [37]	Korea	KNHANES	2016	3086 adults aged 18–64 years (1186 males and 1900 females)	To explore the association between soft-drink consumption and obesity, depression, and subjective health status	One 24-h dietary recall	Quantitative FFQ	24-h dietary recall: estimate total energy and nutrient intakes FFQ: evaluate soft drink intake frequency, serving size, and daily intake	Although various sweetened beverages were provided for sugar intake, the relevance of sugar intake was limited to soft-drink intake alone.
Agogo et al., 2020 [16]	US	NHANES	2003–2004	1605 women aged 12–49 years	To propose three-part regression calibration models to handle excess zeroes, skewness, and heteroscedasticity in adjusting for measurement error in dietary intake data	24-h dietary recall	FFQ	To account for exposure measurement error, several methods were compared: the FFQ method, 24-h recall method, 2-part RC method, and 3-part RC-het-prob method.	NA
Smiliotopoulos et al., 2020 [51]	Greece	Hellenic National Nutrition and Health Survey (HNNHS)	2013–2015	3796 participants aged ≥6 months (1543 males and 2253 females)	To examine the validity of the qualitative FPQ developed to assess the dietary habits of the general population and assess the intake of specific food groups regarding guidelines	Two non-consecutive 24-h dietary recalls	FPQ	Comparison of dietary intake from FPQ with 24-h dietary recall and evaluation of agreement of two methods	This FPQ is a qualitative assessment tool and cannot be used alone for quantitative measurements of energy and macronutrient intakes.
Choi et al., 2019 [38]	Korea	KNHANES	2012–2015	10,460 adults aged 19–64 years (4082 males and 6378 females)	To examine the associations of frequency of consumption of whole fruit and fruit juice with obesity and metabolic syndrome	One 24-h dietary recall	FFQ	24-h dietary recall: estimate energy and macronutrient intakes FFQ: evaluate frequency of whole fruit and fruit juice consumption	The study could not assess whether fruit juices were 100% pure. The frequency of whole fruit and juice consumption relied on a single question, potentially leading to misclassification. Misreporting in dietary intake and energy and macronutrient intakes was possible due to the use of a single 24-h recall that may not represent usual intake.
Bel et al., 2019 [66]	Belgium	Second National Food Consumption Survey	2014–2015	2154 adolescents and adults aged 10–64 years and 992 children aged 3–9 years	To evaluate the habitual food consumption in the general population and to compare it with food-based dietary guidelines and results of the 2004 Food Consumption Survey	Two non-consecutive 24-h dietary recalls (10 to 64 years) and two self-administered non-consecutive one-day food diaries (3 to 9 years)	FFQ	The distribution of habitual food consumption was estimated to combine 24-h recall and FFQ data based on statistical models accounting for within-subject variation.	The difference between habitual consumption and recommendations may be due to under-reporting of food intake.
Ahn et al., 2017 [39]	Korea	KNHANES	2012–2014	10,286 adults aged 19–64 years (3996 males and 6290 females)	To compare the usual nutrient intake in both the semi-quantitative FFQ and 24-h recall methods and determine the association between metabolic syndrome risk and nutrient intake calculated by both methods	24-h dietary recall	Semi-quantitative FFQ	Evaluate the correlation of nutrient intake between 24-h recall and the FFQ and examine the association of metabolic syndrome with nutrient intake from the two methods	The FFQ included meals prepared using different recipes in the same food group; nutrient intakes could be underestimated or overestimated due to variations in seasonings among recipes.
Kim et al., 2017 [14]	Korea	KNHANES	2009	6150 adults aged ≥19 years	To determine whether the lower intakes of yogurt, milk, and calcium were associated with periodontitis	One 24-h dietary recall	FFQ	24-h dietary recall: estimate calcium intake FFQ: evaluate frequency of yogurt and milk intake	Calcium intake in the KNHANES data could only be estimated based on the Korean dietary reference intakes. A single 24-h recall may not accurately reflect habitual calcium intake due to the short survey duration and reliance on individual memory.
Denova-Gutiérre et al., 2016 [71]	Mexico	Mexican National Health and Nutrition Survey (Ensanut 2012)	2012	178 adolescents and 230 adults aged ≥20 years	To assess the validity of a 140-item semiquantitative FFQ	Two non-consecutive 24-h dietary recalls	Semi-quantitative FFQ	Evaluation of agreement of intakes of energy, macro-, and micronutrients from two methods 24-h dietary recall: used as a standard to measure the relative validity of the FFQ	The 24-h dietary recall and FFQ had several sources of error, including reliance on memory and perception of portion size. Reproducibility could not be assessed because the FFQ was not conducted twice.
Loftfield et al., 2016 [17]	US	NHANES	2002–2003, 2005–2006, 2011–2012	6219 adults aged ≥20 years	To estimate usual daily coffee intakes from all coffee-containing beverages, including decaffeinated and regular coffee, based on demographic, socioeconomic, and health-related factors	Two non-consecutive 24-h dietary recalls	FFQ	24-h dietary recall: estimate coffee intake FFQ: evaluate coffee drinking/not drinking	Self-reported dietary assessment methods tended to introduce measurement error. A single 24-h dietary recalls overestimated the proportion of nondrinkers. Caffeine in coffee was not assessed.
Agarwal et al., 2016 [18]	US	NHANES	2001–2002, 2003–2004, 2005–2006, 2007–2008, 2009–2010	24,807 adults aged ≥19 years (12,561 males and 12,246 females)	To examine alcohol’s dose-dependent effects on markers of liver function, as well as to compare the different methods of assessing alcohol intake	Two non-consecutive 24-h dietary recalls	Alcohol consumption questionnaire	Alcohol intake was assessed in three ways: (1) using a single 24-h recall, estimating self-reported alcohol consumption on the day of the recall; (2) using two days of 24-h recalls, estimating long-term intake through the NCI method; and (3) using an alcohol intake questionnaire to quantify annual consumption.	Alcohol intake was often underestimated, which can lead to bias in self-reported intakes.
Choi et al., 2016 [40]	Korea	KNHANES	2007–2009	2510 male adults aged 40–64 years	To investigate the daily intake of vegetables on a national level and its effect on the risk of CHD, as determined by the Framingham Risk Score	24-h dietary recall	FFQ	24-h dietary recall: estimate intakes of carbohydrate, protein, fat, and dietary fiber and dividing into salted and non-salted vegetables FFQ: divide into green and white vegetables	Vegetable intake was only quantitatively evaluated. The 24-h recall method is based on a retrospective method; thus, participant intake may have been difficult to accurately reflect.
Kim et al., 2015 [41]	Korea	KNHANES	2012	7118 participants aged ≥1 year	To examine the prevalence of household food insecurity and compare dietary intake by food security status	One 24-h dietary recall	Semi-quantitative FFQ	24-h dietary recall: estimate intakes of energy and nutrients FFQ: evaluate food and nutrient intakes	The FFQ did not include data on children and older adults.
Chung et al., 2015 [42]	Korea	KNHANES	2007–2011	13,972 adults aged ≥30 years (5432 males and 8540 females)	To examine the association between sugar-sweetened beverage consumption and metabolic syndrome risk factors	One 24-h dietary recall	Qualitative FFQ	24-h dietary recall: estimate nutrient intake FFQ: evaluate frequency of soft drink intake	Soft drink consumption was defined based on a single question, as the FFQ did not include information on other types of sugar-sweetened beverages. Fruit juices were not included as soft drink consumption.
Engle-Stone et al., 2015 [34]	Cameroon	National dietary survey	2009	24-h dietary recall: 912 females aged 15–49 years and 882 children aged 12–59 months FFQ: 901 females aged 15–49 years and 901 children aged 12–59 months	To compare apparent consumption of fortifiable foods estimated from the Third Cameroon Household Survey (ECAM3) conducted in 2007 with the results of a national dietary survey using the food frequency questionnaire and 24-h recall methods	One 24-h dietary recall	FFQ	Comparison FFQ and 24-h recall data with ECAM3 data24-h dietary recall: estimate amount of four fortifiable foods consumed (refined vegetable oil, wheat flour, sugar, and bouillon cube) FFQ: evaluate frequency of consumption of four fortifiable foods	Total nutrient intakes were not compared.
Zhu et al., 2015 [19]	US	NHANES	2003–2004, 2005–2006	5124 children aged 2–18 years	To examine the associations between yogurt consumption, diet quality, and metabolic profiles in children	At least one 24-h dietary recall	Qualitative FFQ	24-h dietary recall: estimate energy intake and assessment of diet quality FFQ: evaluate frequency of yogurt consumption	The FFQ was not quantitative and did not differentiate between type of yogurt (plain yogurt, probiotic yogurt, etc.).
Parackal et al., 2015 [63]	New Zealand	National Nutrition Survey (NZANS)	2008–2009	2995 adolescent and adults aged ≥15 years (1308 males and 1687 females)	To investigate similarities and differences in dietary habits, nutrient intakes, and health outcomes by ethnic group and examine differences within the Asian subgroups based on duration of residence	At least one 24-h dietary recall	Dietary habits questionnaire	24-h dietary recall: estimate nutrient intakes Dietary habits questionnaire: evaluate frequency of food intake	A single 24-h recall did not capture usual intake and could underreport.
Eisinger-Watzl et al., 2015 [61]	Germany	German National Nutrition Survey II	2005–2007	9968 participants aged 14–80 years	To compare the updated version of the diet history method and the 24-h recall method	Two 24-h dietary recalls	Semi-quantitative diet history	Evaluate the agreement of dietary intake of the two methods (24-h recalls and diet history)	NA
Park et al., 2015 [43]	Korea	KNHANES	2007–2009, 2010–2012	27,656 adults aged ≥20 years (1308 males and 1687 females)	To assess whether the intake of vitamin A (including β-carotene), vitamin C, fruits, or vegetables was negatively associated with metabolic syndrome	24-h dietary recall	Semi-quantitative FFQ	24-h dietary recall: estimate energy and nutrient intakes FFQ: evaluate frequency of intake of green vegetables, white vegetables, and fruits	The 24-h recall method is based on memory and may lead to underestimation or overestimation; thus, bias may have occurred in the determination of typical nutrient intakes. The FFQ could have caused misclassification, potentially overestimating fruit and vegetable intake.
Kim et al., 2014 [44]	Korea	KNHANES	2008–2010	14,428 adults aged 20–64 years (5917 males and 8511 females)	To provide useful insights into plain water intake based on lifestyle, anthropometric, and dietary characteristics	One 24-h dietary recall	FFQ	24-h dietary recall: estimate food and nutrient intakes and diet quality FFQ: evaluate frequency of beverage intake, such as coffee and tea	The periods for which the plain water intake and 24-h recall methods were evaluated differed. A single 24-h recall did not provide an accurate estimate of usual intake.
Kim et al., 2014 [45]	Korea	KNHANES	2007–2011	17,953 adults aged 19–65 years	To investigate the relationship between coffee consumption and metabolic syndrome and its components	One 24-h dietary recall	Qualitative FFQ	24-h dietary recall: estimate intakes of sugar/syrup, coffee creamer, and milk and classification of coffee drinkers FFQ: classification of coffee drinkers	Using only one 24-h recall may have led to an underestimate of consumption.
Patel et al., 2014 [20]	US	NHANES	2003–2010	4525 males ≥40 years	To evaluate prostate-specific antigen (PSA) levels with fish consumption (the primary source of n-3 PUFAs) and calculated PUFA intake	Two 24-h dietary recalls	FFQ	24-h dietary recall: estimate EPA and DHA FFQ: evaluate specific types of fish consumed	Both the FFQ and 24-h dietary recall may have limitations because they capture relatively short-term exposure.
Lee et al., 2014 [46]	Korea	KNHANES	2007–2009	7173 adults aged 19–64 years (3400 males and 3773 females)	To investigate the association between dairy products and calcium intake and obesity	One 24-h dietary recall	Qualitative FFQ	24-h dietary recall: estimate dairy product intake and intakes of total calcium, non-dairy calcium, and dairy calcium FFQ: evaluate frequency of milk and yogurt intake	NA
Yang et al., 2014 [47]	Korea	KNHANES	2008–2010	2305 males aged 50–79 years	To investigate general determinants and dietary factors that influenced bone mineral density	One 24-h dietary recall	Qualitative FFQ	24-h dietary recall: estimate nutrient intakes FFQ: evaluate frequency of food intakes	Nutrient intake was estimated using a single-day 24-h recall. Because KNHANES does not ask about dietary supplement intake, calcium and vitamin D dietary supplement intake was not considered in the overall intake.
Kim et al., 2013 [48]	Korea	KNHANES	2007–2008	500 hypertensive (269 males and 231 females) and 4567 normotensive (1749 males and 2818 females) participants aged ≥20 years	To examine the hypothesis that adherence to the JNC-7 guidelines is associated with hypertension	One 24-h dietary recall	Non-quantitative FFQ	24-h dietary recall: estimate energy and nutrient intakes FFQ: evaluate frequency of intake by food (group)	There could have been measurement errors in the 24-h dietary recalls or FFQs.
Bjermo et al., 2013 [52]	Sweden	Riksmaten 2010–2011	2010–2011	273 participants aged 18–80 years (128 males and 145 females)	To examine the bodily burden of lead, mercury, and cadmium in blood and the association between blood levels, diet, and other lifestyle factors	Four-day food records	FFQ; questions regarding less frequency consumed items (e.g., consumption frequency of different classes of fish and meat)	Food records: estimate alcohol, vegetable, and meat intakes FFQ: evaluate less frequently consumed foods (game and fish)	NA
Kappeler et al., 2013 [21]	US	NHANES	1986–2010	17,611 participants ≥18 years (8239 males and 9372 females)	To evaluate the association of meat intake and healthy eating index with total mortality, cancer, and cardiovascular disease	One 24-h dietary recall	Qualitative FFQ	24-h dietary recall: calculate the healthy eating index score FFQ: evaluate frequency of food intake	NHANES III used an FFQ to assess food consumption, but it could not determine portion sizes. The healthy eating index scores relied on a single 24-h dietary recall.
Bjermo et al., 2013 [53]	Sweden	Riksmaten 2010-2011	2010–2011	246 adults with an average age of 50 (113 males and 133 females)	To investigate the bodily burden of several POPs and their association with diet and other lifestyle factors	Four consecutive days food records	FFQ	Food records: estimate intakes of fat from various animal food groups (i.e., fish, meat, egg, sausage, offal, and poultry) and pollutant concentrations FFQ: evaluate less frequently consumed food items (e.g., fish, meat)	Although the POPs serum concentrations represent long-term exposure prior to blood sampling, dietary patterns were recorded for only four days on one occasion. Intake of less commonly eaten fish may have been underestimated in the food records.
Doidge et al., 2012 [33]	Australia	National Nutrition Survey (NNS)	1995	9096 participants aged ≥12 years	To describe the pattern of dietary consumption in Australians and assess the extent to which the population met the national recommendations	At least one 24-h dietary recall	FFQ	Estimate distribution of dairy consumption by age and gender using FFQ and 24-h recall data 24-h dietary recall: estimation of food and nutrient intakes FFQ: evaluate frequency of consumption of foods and supplements	Grouping all dairy foods together assumed that participants consumed milk-, cheese-, and yogurt-based products in the same relative proportions.
Hoffmire et al., 2012 [22]	US	NHANES	2005–2008	9276 adults aged ≥20 years	To assess the association between fish consumption and depressive symptoms	Two non-consecutive 24-h dietary recalls	FFQ	24-h dietary recall: estimate energy and nutrient intakes, including EPA + DHA FFQ: determining the intake of different types of fish	Both FFQ and dietary recall captured short-term fish intake and were not suitable for quantifying more chronic (over years) intake.
Lee et al., 2011 [57]	Taiwan	Elderly Nutrition and Health Survey in Taiwan (NAHSIT 1999/2000)	1999–2000	1743 older adults aged ≥ 65 years (860 males and 883 females)	To assess the relative predictive ability for mortality of the Overall Dietary Index—Revised (ODI-R) and the Dietary Diversity Score (DDS).	At least one 24-h dietary recall	FFQ	Age- and gender-specific serving sizes for each food group obtained from the 24-h recall were applied to the FFQ. 24-h dietary recall: derive a DDS FFQ: calculate the number of food group servings and nutrient intakes required for the ODI-R score	The dietary information was collected based on food-nutrient categories rather than specific individual foods, and the DDS did not distinguish between soy and animal-derived foods.
Wolfe et al., 2011 [23]	US	National Health and Nutrition Evaluation Survey Epidemiologic Follow-up Study (NHEFS)	1971–1984 (follow-up period: 1982–1984)	4856 adults aged 25–74 years (1947 males and 2909 females)	To examine the effect of dietary protein and protein-rich food on severely depressed mood	One 24-h dietary recall	FFQ	24-h dietary recall: estimate nutrient intake FFQ: evaluate frequency of consuming high-protein food	Food intake was assessed only once at baseline, potentially introducing measurement error due to self-reporting. Protein sources (animal and vegetable proteins) were not differentiated.
Tran et al., 2010 [24]	US	NHANES	2003–2004	5306 participants (1019 children aged 3–12 years, 1201 adolescents aged 13–19 years (561 males and 640 females), 3086 adults aged ≥20 years (1408 males and 1678 females))	To evaluate the relationship between dietary acrylamide (AA) and hemoglobin adducts	At least one 24-h dietary recall	Qualitative FFQ	The FFQ data and the quantities of food consumed per eating occasion or day, assessed from the 24-h dietary recall, were combined with AA concentration data to estimate long-term (usual) AA dietary exposure.	A potential limitation of using a self-reporting tool like the FFQ is the possibility of reporting bias, which can affect the accuracy of intake measurements.
Haftenberger et al., 2010 [62]	Germany	German National Nutrition Monitoring (NEMONIT)	2008–2011	161 adults aged 18–79 years (82 males and 79 females)	To examine the relative validity of an FFQ developed for use in the German Health Examination Survey for Adults 2008-2011	Two 24-h dietary recalls (in four consecutive waves)	Semi-quantitative FFQ	Comparison of food intake from FFQ with 24-h dietary recall and ranking evaluation of agreement of food groups from two methods	Both the FFQ and the 24-h dietary recall shared similar error sources, including the reliance on memory and the perception of portion sizes. The FFQ depended on participants’ capability to quantify how much they ate of individual foods and mixed dishes.
Lee et al., 2010 [58]	Taiwan	Elderly Nutrition and Health Survey in Taiwan (NAHSIT 1999/2000)	1999–2000	1410 older adults aged ≥65 years (729 males and 681 females)	To examine chewing ability and survival in older adults and consider any interaction with the metabolic syndrome	24-h dietary recall	FFQ	24-h dietary recall: derive the Dietary Diversity Score FFQ: evaluate frequency of food intake	NA
Temme et al., 2010 [67]	Belgium	Belgian National Food Consumption Survey (BNFCS)	2004	3245 participants aged ≥15 years (1623 males and 1622 females)	To evaluate current dietary energy and macronutrient intakes and compare them with the national dietary guidelines and to describe the main food sources of the macronutrients	Two non-consecutive 24-h dietary recalls	FFQ	NA	NA
Bailey et al., 2010 [25]	US	NHANES	2003–2004, 2005–2006	11,462 participants aged ≥ 14 years (5910 males and 5552 females)	To combine data on dietary folate and folic acid from dietary supplements with the use of the bias-corrected best power method to adjust for within-person variability	Two non-consecutive 24-h dietary recalls	Dietary supplement questionnaire	24-h dietary recall: estimate dietary folate intake FFQ: evaluate average daily intake of folic acid derived from supplements	Dietary intake estimates were adjusted for within-individual variation to reflect usual intake. Folate in food estimates and folic acid content in dietary supplements were primarily based on label values.
Fraser et al., 2009 [26]	US	NHANES	2003–2004	PBDE data: 2040 (994 males and 1046 females), 24-h recall data: 1971 (964 males and 1007 females), FFQ data 1536 (707 males and 829 females)	To evaluate the dietary contribution to PBDE bodily burdens in the United States by linking serum levels to food intake.	Two non-consecutive 24-h dietary recalls	Qualitative FFQ	24-h dietary recall: estimate absolute food and nutrient intakesFFQ: evaluate average intakes of food over a longer period of time	Although 24-h food records aimed for accuracy over a short period, they did not fully represent usual diets and could lead to exposure misclassification when long-term diet was more appropriate. However, the FFQ estimated food consumption over the previous year.
de Boer et al., 2009 [27]	US	NHANES	1988–1994	15,513 adults aged ≥20 years	To examine nutritional variables and cardiovascular risk factors regarding circulating serum phosphorus concentrations	24-h dietary recall	FFQ	24-h dietary recall: estimate intakes of total energy, macronutrients, phosphorus, and alcohol FFQ: evaluate food intakes	Dietary surveys were imprecise.
Vandevijvere et al., 2009 [68]	Belgium	Belgian National Food Consumption Survey (BNFCS)	2004	3245 adolescents and adults aged ≥15 years	To evaluate the gap between food-based dietary guidelines and usual food consumption	Two non-consecutive 24-h dietary recalls	FFQ	FFQ data were used to determine the percentage of individuals consuming specific foods daily. 24-h dietary recall: estimate food groups intake FFQ: assess the percentage of persons who consume certain foods on a daily basis	NA
Yu et al., 2008 [59]	Taiwan	Nutrition and Health Survey in Taiwan (NAHSIT)	1993–1996	2176 adults aged 19–64 years (987 males and 1189 females)	To clarify the dietary factors associated with hyperuricemia	One 24-h dietary recall	Qualitative FFQ for food groups and FFQ for alcohol consumption	The associations between food intakes derived from each dietary survey and hyperuricemia were examined. 24-h dietary recall: estimate energy, nutrient, and food group intakeFFQ: evaluate food group	The purine content of food was not included in the Nutrient Composition Data Bank for Foods of the Taiwan Area database.
Laroche et al., 2007 [28]	US	NHANES	1988–1994	6660 adults aged 17–65 years (52% females)	To compare dietary fat intake between adults with and without minor children in the home	One 24-h dietary recall	Qualitative FFQ	24-h dietary recall: estimate intake of total fat, saturated fat, and kilocalories FFQ: evaluate frequency of high-fat foods	Only one 24-h dietary recall was available per individual. Food frequency analysis was restricted to NHANES dataset questions, and some food groups had options with variable fat content.
Labadarios et al., 2005 [69]	Republic of South Africa	National Food Consumption Survey (NFCS)	1999	2894 children aged 1–9 years	To determine the nutrient intake and anthropometric status of children and factors that influence their dietary intake	Three separate 24-h dietary recalls	Qualitative FFQ	Comparison of FFQ data with 24-h dietary recall data 24-h dietary recall: evaluate food intake FFQ: estimation of energy, nutrient, and food intakes	NA
Andersen et al., 2003 [64]	Norway	NA	1999	64 infants aged 12 months (26 boys and 37 girls)	To assess the validity of a semi-quantitative FFQ used in a large nation-wide dietary survey among 12-month-old Norwegian infants	Seven-day weighed food records	Semi-quantitative FFQ	Evaluate the agreement of the two methods (food records and FFQ) at the category level	The inclusion of overly large portion sizes in the FFQ can be problematic, as infants around 12 months of age may taste a variety of foods without consuming real portions.
Nelson et al., 2002 [29]	US	NHANES	1988–1994	1480 adults ≥ 17 years (640 males and 840 females)	To describe diet and exercise practices of U.S. adults with type 2 diabetes	One 24-h dietary recall	FFQ	24-h dietary recall: calculate percentage of daily calories from total fat and saturated fat FFQ: create a combined variable for daily fruit and vegetable intake	The FFQ and 24-h recall data were self-reported.
Heller et al., 2001 [30]	US	NHANES	1988–1944	19,668 participants aged ≥2 years	To investigate the associations between sugared soda consumption and caries	One 24-h dietary recall	Qualitative FFQ	24-h dietary recall: evaluate frequency and amount of sugared soda intake FFQ: evaluate frequency of sugared soda (12 years and older only)	The FFQ data relied on participant memory for the previous month’s diet and did not include food quantity information. The 24-h recall recorded only single-day data and there may have been recall bias (e.g., participants may have underestimated sugared soda consumption).
Smit et al., 2001 [31]	US	NHANES	1988–1944	10,623 adults aged 20–59 years	To examine dietary intake and nutritional status of marijuana users and non-current marijuana users	One 24-h dietary recall	Qualitative FFQ	24-h dietary recall: estimate nutrient intake FFQ: evaluate frequency of food (group) intake	The 24-h recall did not capture typical or individual intake and excluded supplements. The FFQ lacked quantitative nutrient details.
Song et al., 2000 [32]	US	NHANES	1988–1944	27,378 participants ≥ 2 months (13,321 males and 14,057 females)	(1) To assess the nutritional significance of eggs in the American diet (2) To estimate the association between egg consumption and serum cholesterol concentration	One 24-h dietary recall	FFQ	24-h dietary recall: estimate nutrient intake FFQ: evaluate frequency of egg intake	NA

NA, not applicable; FFQ, food frequency questionnaire; FPQ, food propensity questionnaire; NHANES, National Health and Nutrition Examination Survey; KNHANES, Korea National Health and Nutrition Examination Survey; NHEFS, a study of adults who originally participated in the NHANES I.

## Data Availability

Data are contained within the article.
